# Comparison of the air kerma standards of ARPANSA, Australia, and the IAEA in RQR, RQA, and RQT diagnostic X-ray beams

**DOI:** 10.1007/s13246-025-01570-w

**Published:** 2025-05-28

**Authors:** Kam L. Lee, Zakithi Msimang, Duncan Butler, Peter Thomas, Joao Cardoso

**Affiliations:** 1 Australian Radiation Protection and Nuclear Safety Agency, 619 Lower Plenty Road, Yallambie, VIC 3085 Australia; 2 Division of Human Health, Department of Nuclear Sciences and Applications, Vienna International Centre, International Atomic Energy Agency, PO Box 100, 1400 Vienna, Austria

**Keywords:** Calibration, Bilateral comparison, RQR, RQA, RQT

## Abstract

A comparison of air kerma standards between the Australian Radiation Protection and Nuclear Safety Agency (ARPANSA) and the International Atomic Energy Agency (IAEA) was carried out for the RQR, RQA and RQT radiation qualities (as prescribed by IEC 61267 or IAEA TRS 457) using two ionisation chambers as transfer standards. The ratios between the IAEA and ARPANSA calibration coefficients ranged between 0.993 and 0.997 for RQR beams, 0.990 and 0.999 for RQA beams, and 0.993 to 0.994 for RQT beams. The relative expanded uncertainty for IAEA and ARPANSA was 1.2 and 1.4% respectively, and the comparison results agree at around the level of, or better than, the combined standard uncertainty of 0.74% for all the beams.

## Introduction

The role of ionising radiation in medical imaging is pivotal in providing diagnostic information to answer clinical questions. On the other hand, the deleterious effects (tissue reactions, hereditary and stochastic) of ionising radiation are well known [1]. Therefore, efforts, such as dose optimization and QA programs, are made to minimise the risks associated with using ionising radiation in medical imaging whilst ensuring clinical tasks are fulfilled. In particular, national diagnostic reference levels have been established and quality assurance standards procedures published and implemented for various modalities [2–7].

A core element in all these efforts is the collection of radiation dose metrics. Whether it is simply recording kerma-area product (KAP) information displayed by the system or from the Digital Imaging and Communications in Medicine (DICOM) header or actually measuring the air kerma, radiation measurement devices are involved. Hence, the accuracy and traceability of these radiation measurement devices are of paramount importance. This is reflected in the recent establishment of the IAEA coordinated research project [8] and the Traceability in Medical X-ray Imaging Dosimetry (TraMeXI) project; an international consortium of metrology laboratories [9]. The Code for Radiation Protection in Medical Exposure (RPS C-5), published by ARPANSA, states that the Responsible Person must ensure that “calibration of all dosimeters used for dosimetry of patients and for the calibration of sources is traceable to a standards dosimetry laboratory, and is undertaken at intervals approved by the relevant regulatory authority” [10]. A similar requirement for calibration of compliance testing equipment is included in the Standard for Radiation Safety and Performance Testing of Diagnostic Imaging Apparatus (RPS S-2) [11].

Globally there are around 20 national primary standard dosimetry laboratories and many secondary standard dosimetry laboratories that provide calibration services, including services for diagnostic equipment. These laboratories regularly compare their measurements of air kerma and absorbed dose under internationally agreed programmes [12] arranged through the Bureau International des Poids et Mesures (BIPM). The BIPM maintains a public database of the results: the Key Comparison Database (KCDB) [13] which currently shows results for air kerma for ARPANSA and IAEA in low energy X-rays (10–50 kV) and ARPANSA in medium (50–250 kV) X-rays (these comparisons use the identifiers BIPM.RI(I)-K2 and BIPM.RI(I)-K3 in the KCDB). However, the X-ray beam qualities used in the BIPM comparisons are different to the diagnostic beam qualities used to calibrate and assess diagnostic equipment. They cover the same energy range but the filtration and beam qualities are different. Hence the motivation to conduct a comparison using diagnostic beams.

This paper reports a bilateral comparison between the ARPANSA and the IAEA for the diagnostic beam qualities RQR, RQA and RQT, where the beams in both laboratories are as prescribed by IEC 61267 or IAEA TRS 457 [14, 15]. These beam qualities are directly relevant to clinical applications, with the RQR series designed to match the incident beams commonly used in general radiography, fluoroscopy and dental applications, while the RQA series is for beams exiting the patient and RQT is for computed tomography [14, 15]. The bilateral comparison between ARPANSA and IAEA provides measurement confidence for the capabilities of these laboratories and supports the traceability provided to their clients for clinical measurements.

## Materials and methods

### Equipment

The comparison was conducted by comparing the results of the calibrations of two ionisation chambers (“transfer standards”) in the X-ray calibration laboratories of the two institutes. The technical details for the ionisation chambers and X-ray equipment are listed in Tables 1 and 2 respectively. The Exradin A3 is a spherical ionisation chamber and the PTW 34069 is a plane parallel ionisation chamber. Both chambers were supplied by the IAEA. A positive polarising voltage was applied to the collector in the chamber.

**Table 1:** Technical details of the comparison chambers [16, 17]

Type	Reference point	Nominal volume (cm^3^)	Active volume dimensions (mm)	Polarising voltage (V)
Exradin A3, S/N XR072321	Chamber Centre	3.6	Radius: 9.7	+ 300
PTW 34069, S/N 421	Chamber Centre	6	Radius: 15.2 Depth: 8.42	+ 300

**Table 2:** X-ray equipment technical details

Laboratory	Manufacturer	X-ray tube model	Anode material	Anode angle (°)	Nominal spot size (mm)
IAEA	General Electrics (GE)	Isovolt Titan E with MXR 160	Tungsten	20	5.5
ARPANSA	Gulmay Limited	Comet MXR-320/26–90	Tungsten	20	5.5

### Comparison protocol

Prior to the comparison, the RQR, RQA and RQT series of beam qualities were established as per the requirements prescribed by IEC and IAEA [14, 15]. IAEA followed the methodology described in IAEA TRS 457 [15] to establish these beam qualities whilst ARPANSA used an in-house developed methodology to establish these beam qualities [18]. The transfer standard chambers were first calibrated at the IAEA dosimetry laboratory (DOL) according to the IAEA calibration procedures (see [19] for details).

The IAEA maintains secondary standards for the determination of air kerma for the X ray beam qualities used in diagnostic radiology. The ionization chambers of the IAEA are traceable to the Physikalisch-Technische Bundesanstalt (PTB). The details of the radiation beam qualities are given in Tables 5, 6, 7.

The transfer standard chambers were subsequently sent to ARPANSA for calibration. ARPANSA maintains a medium energy (50–250 kV) free-air chamber (MEFAC, see [20] for details) as their primary standard. Details of the ARPANSA calibration procedures can be found in [18]. Finally, the chambers were sent back to IAEA DOL for another calibration.

The calibration coefficients for the transfer standard chambers were determined, corrected to standard conditions listed in the Reference conditions section. The average of the two IAEA calibration coefficients (${N }_{K}^{IAEA}$) was used in this comparison exercise. The calibration was performed using the substitution method [15] and monitor chambers were used for normalization of the X-ray beam output in both laboratories. For a transfer standard chamber, the calibration coefficient ${N}_{K}$ at a particular beam quality q was given by:

1 $${N}_{K,q}={N}_{K,q}^{mon}\left(\frac{{k}_{TP}^{mon}}{{k}_{TP}^{cham}}\right)\left(\frac{m}{M}\right)$$

where ${N}_{K,q}^{mon}$ is the calibration coefficient of the monitor chamber, ${k}_{TP}^{mon}$ is the air density correction for the monitor chamber, ${k}_{TP}^{cham}$ is the air density correction for the transfer standard chamber, m is the reading from the monitor chamber and M is the reading from the transfer standard chamber.

This comparison protocol complies with the IAEA technical protocol of the diagnostic radiology comparison [21]. The ratio of calibration coefficients (*R*) was calculated for each beam quality and is defined as

2 $$R=\frac{{N}_{K,q}^{ARPANSA}}{{N}_{K,q}^{IAEA}}$$

where ${N }_{K,q}^{ARPANSA}$ is the ARPANSA calibration coefficient at beam quality q and ${N }_{K,q}^{IAEA}$ is the average IAEA calibration coefficient at beam quality q.

The Exradin A3 chamber was used as the transfer standard for RQR and RQT calibrations while the PTW 34069 chamber was used as the transfer standard for RQA calibrations.

### Reference conditions

The focus to calibration point distance and radiation field diameter used in both laboratories were 1000 and 100 mm respectively. The orientation mark on the stem of each chamber was aligned towards the X ray tube. The X-ray tube currents used at ARPANSA were 10 mA for RQR series and 20 mA for RQA and RQT series. Table 3 lists the tube currents used in the IAEA laboratory.

**Table 3:** Tube currents used by the IAEA laboratory

RQR radiation quality	Tube current (mA)	RQA radiation quality	Tube current (mA)	RQT radiation quality	Tube current (mA)
2	32.4	2	14.4		
3	18.4	3	20.9		
4	13.6	4	24.3		
5	10.7	5	21.6		
6	8.9	6	19.0		
7	7.6	7	15.5		
8	6.4	8	13.5	8	15.2
9	5.0	9	9.9	9	10.9
10	3.7	10	6.1	10	7.1

The calibration coefficients provided by each laboratory were corrected to the standard ambient conditions: temperature = 293.15 K, pressure = 101.325 kPa, relative humidity in air between 30 and 70%.

### Uncertainty

The relative uncertainties (*u*_i_) for the physical constants for the ARPANSA MEFAC are listed in Table 4. In Table 4, *W*_air_ represents the work function of air, *e* represents the electronic charge and *W*_*air*_*/e* represents the average energy necessary to produce an electron–ion pair in air.

**Table 4:** Physical constants and their relative uncertainties

Constant	Value	*u*_i_ (%)
Air density	1.2047 kg/m^3^	0.01 [22]
*W*_air_/*e*	33.97 J/C	0.35 [23]

Following the Joint Committee for Guides in Metrology (JCGM) principles [24], the uncertainty budgets were formulated by each laboratory. Uncertainty components were categorised into type A and type B where type A uncertainties were determined statistically from measurements while type B uncertainties were derived from other sources, such as past assessments of variability or uncertainties stated in reference data. Standard uncertainties were summed in quadrature to derive the combined standard uncertainty. The combined relative expanded uncertainty was obtained by multiplying the combined standard uncertainty by the coverage factor (k). The coverage factor was chosen as two, which corresponds to a level of confidence of approximately 95%.

## Results

### Beam quality comparison

The RQR, RQA and RQT series of radiation qualities established in each laboratory are listed in Tables 5, 6, 7.

**Table 5:** RQR radiation qualities comparisons

Radiation quality	Tube voltage (kV)	Air kerma rate IAEA (mGy/min)	Air kerma rate ARPANSA (mGy/min)	First HVL IEC (mm Al)	First HVL IAEA (mm Al)	First HVL ARPANSA (mm Al)
RQR-2	40	42	27	1.42	1.42	1.40
RQR-3	50	42	44	1.78	1.78	1.76
RQR-4	60	38	57	2.19	2.18	2.21
RQR-5	70	38	72	2.58	2.57	2.56
RQR-6	80	41	42	3.01	3.00	2.99
RQR-7	90	41	52	3.48	3.58	3.42
RQR-8	100	41	30	3.97	4.02	4.00
RQR-9	120	41	154	5.00	5.04	5.03
RQR-10	150	40	208	6.57	6.71	6.62

*HVL* half-value layer, *IEC* IEC 61267

**Table 6:** RQA radiation qualities comparisons

Radiation quality	Tube voltage (kV)	Air kerma rate IAEA (mGy/min)	Air kerma rate ARPANSA (mGy/min)	First HVL IEC (mm Al)	First HVL IAEA (mm Al)	First HVL ARPANSA (mm Al)
RQA-2	40	3.1	6.6	2.2	2.21	2.28
RQA-3	50	3.1	3.9	3.8	3.77	3.99
RQA-4	60	3.0	3.3	5.4	5.38	5.49
RQA-5	70	3.0	3.5	6.8	6.89	7.00
RQA-6	80	3.1	7.2	8.2	8.24	8.38
RQA-7	90	3.1	8.9	9.2	9.26	9.47
RQA-8	100	3.1	10.5	10.1	10.24	10.44
RQA-9	120	3.1	14.7	11.6	11.64	11.96
RQA-10	150	3.1	24.5	13.3	13.40	13.72

*HVL* half-value layer, *IEC* IEC 61267

**Table 7:** RQT radiation qualities comparisons

Radiation quality	Tube voltage (kV)	Air kerma rate IAEA (mGy/min)	Air kerma rate ARPANSA (mGy/min)	First HVL IEC (mm Al)	First HVL IAEA (mm Al)	First HVL ARPANSA (mm Al)
RQT-8	100	41	54	6.9	7.02	6.92
RQT-9	120	41	72	8.4	8.47	8.55
RQT-10	150	41	111	10.1	10.26	10.26

*HVL* half-value layer, *IEC* IEC 61267

### Calibration comparisons

Tables 8, 9, 10 show the IAEA and ARPANSA calibration coefficients, the differences in the pre- and post-transfer IAEA calibration coefficients, the averages of the pre- and post-transfer IAEA calibration coefficients and the calibration coefficient ratios (*R*) for the radiation qualities RQR, RQA and RQT.

**Table 8:** RQR calibration coefficient comparisons

Radiation quality	Pre-transfer ${N}_{k}^{IAEA}$ (mGy/nC)	Post-transfer ${N}_{k}^{IAEA}$ (mGy/nC)	Difference (%)	Average ${N}_{k}^{IAEA}$ (mGy/nC)	${N}_{k}^{ARPANSA}$ (mGy/nC)	*R*
RQR-2	8.206	8.205	− 0.01	8.206	8.163	0.995
RQR-3	8.153	8.155	0.02	8.154	8.112	0.995
RQR-4	8.124	8.122	− 0.02	8.123	8.069	0.993
RQR-5	8.104	8.103	− 0.01	8.104	8.060	0.995
RQR-6	8.093	8.091	− 0.02	8.092	8.052	0.995
RQR-7	8.092	8.090	− 0.02	8.091	8.044	0.994
RQR-8	8.088	8.088	0.00	8.088	8.045	0.995
RQR-9	8.082	8.081	− 0.01	8.082	8.050	0.996
RQR-10	8.084	8.086	0.02	8.085	8.057	0.997

**Table 9:** RQA calibration coefficient comparisons

Radiation quality	Pre-transfer ${N}_{k}^{IAEA}$ (mGy/nC)	Post-transfer ${N}_{k}^{IAEA}$ (mGy/nC)	Difference (%)	Average ${N}_{k}^{IAEA}$ (mGy/nC)	${N}_{k}^{ARPANSA}$ (mGy/nC)	*R*
RQA-2	4.212	4.215	0.07	4.214	4.208	0.999
RQA-3	4.143	4.149	0.14	4.146	4.132	0.997
RQA-4	4.113	4.118	0.12	4.116	4.100	0.996
RQA-5	4.105	4.110	0.12	4.108	4.083	0.994
RQA-6	4.097	4.102	0.12	4.100	4.068	0.992
RQA-7	4.087	4.091	0.10	4.089	4.072	0.996
RQA-8	4.087	4.094	0.17	4.091	4.063	0.993
RQA-9	4.079	4.084	0.12	4.082	4.041	0.990
RQA-10	4.034	4.040	0.15	4.037	4.011	0.994

**Table 10:** RQT calibration coefficient comparisons

Radiation quality	Pre-transfer ${N}_{k}^{IAEA}$ (mGy/nC)	Post-transfer ${N}_{k}^{IAEA}$ (mGy/nC)	Difference (%)	Average ${N}_{k}^{IAEA}$ (mGy/nC)	${N}_{k}^{ARPANSA}$ (mGy/nC)	*R*
RQT-8	8.075	8.074	− 0.01	8.075	8.016	0.993
RQT-9	8.083	8.084	0.01	8.084	8.038	0.994
RQT-10	8.102	8.109	0.09	8.106	8.057	0.994

### Uncertainty budgets

Tables 11, 12 detail the IAEA and ARPANSA uncertainty budgets. Both laboratories followed the Guide to the Expression of Uncertainty in Measurement (GUM) principles in deriving the uncertainty budgets [24].

**Table 11:** IAEA measurement uncertainties

Air kerma rate	Uncertainty (%)	
Using DOL electrometer	Type A	Type B
Step 1: Reference standard (RS)		
Calibration from BIPM/PSDL		0.48
Long term stability of the secondary standard		0.12
Spectral difference PSDL/IAEA		0.06
Current measurement—RS	0.05	0.10
Temperature and pressure correction—RS		0.05
Current measurement—Monitor	0.05	0.10
Temperature and pressure correction—Monitor		0.05
Relative combined standard uncertainty in Step 1	0.07	0.52
Step 2: Instrument to be calibrated		
Current measurement—user chamber	0.06	0.10
Temperature and pressure correction—user chamber		0.05
Current measurement—monitor	0.05	0.10
Temperature and pressure correction—monitor		0.05
Stability of the monitor chamber		0.09
Difference in radial non-uniformity of the beam		0.09
Chamber positioning		0.01
Relative combined standard uncertainty in Step 2	0.08	0.20
Relative combined standard uncertainty (Steps 1 + 2)	0.11	0.55
Standard combined uncertainty (*k* = 1)	0.56	
Combined relative expanded uncertainty (*k* = 2)	**1.2**

**Table 12:** ARPANSA measurement uncertainties

Uncertainty component	Standard uncertainty (%)	
	Type A	Type B
Step 1: Reference standard		
ARPANSA medium-energy free air chamber^*a*^	0.05	0.27
Electrometer random effects	0.03	–
Electrometer linearity (included below)		
Position of standard (included below)		
*W*_*air*_*/e* Physical constants		0.35
Relative combined standard uncertainty in step 1	0.06	0.44
Step2: Instrument to be calibrated		
Electrometer linearity	0.05	–
Temperature and pressure correction for transfer chamber	0.03	–
Recombination losses	0.1	
Reproducibility (dominated by temperature fluctuations)^*b*^	0.50	–
Position of transfer standard relative to reference standard	–	0.10
Transfer standard rotation and tilting	–	0.14
Relative combined standard uncertainty in step 2	0.51	0.17
Relative combined standard uncertainty (Steps 1 + 2)	0.52	0.47
Standard combined uncertainty (*k* = 1)	0.70	
Combined relative expanded uncertainty (*k* = 2)	**1.4**	

^a^Uncertainties associated with the corrections and measurements for the primary standard, including those for temperature, pressure and humidity. See reference [20] for details

^b^The reproducibility includes several effects but is dominated by temperature fluctuations, the effect of which are not completely removed by temperature measurements. See comments in text

## Discussion

Tables 5, 6, 7 compare the first half-value layers measured at the IAEA and ARPANSA laboratories with the IEC standard [14]. These comparison results verify that the RQR, RQA and RQT series of radiation qualities were properly established in both laboratories.

From Tables 8 and 9, the difference in IAEA calibration coefficients before and after the chamber transfer was less than 0.09% for the Exradin A3 chamber. From Table 10, the difference was less than 0.17% for the PTW 34069 chamber. These variations are smaller than the other uncertainties in the intercomparison. Hence the stabilities of the chambers were acceptable. The minimum calibration coefficient ratios for RQR, RQA and RQT were 0.993, 0.990 and 0.993 respectively. This shows that the calibration coefficients reported by both laboratories were in close agreement.

Uncertainties for the ARPANSA MEFAC primary standard were published in the BIPM.RI(I)-K3 report [20] which covered the diagnostic beam energies. Since the same electrometer was used to measure the primary standard and the transfer standard, the absolute calibration of the electrometer in both cases was correlated. Hence, only Type A and non-linearity contributions were accounted for in the ARPANSA uncertainty budget (Table 12). Similarly for temperature and pressure measurements, only non-systematic contributions were accounted for in the ARPANSA uncertainty budget.

The response of the stems of the transfer standards was not investigated: we assume any stem effect would cancel as both laboratories used the same field size, 10 cm. Recombination losses were not corrected. Measurements at the highest dose rate ARPANSA beams (208 mGy/min for RQR-10 for the A3 chamber, and 24.5 mGy/min for RQA-10 for the PTW chamber) indicated recombination losses of less than 0.1% and this has been included as an uncertainty in Table 12. Polarity effects were also ignored as the comparison took place at one bias voltage used at both laboratories.

To correct for any variations in the X-ray tube output, a transmissive monitor chamber was used. However, this caused an additional uncertainty component, included in the ARPANSA uncertainty budget as “Reproducibility” under Step 2 in Table 12, due to temperature fluctuations during the measurements which are only partially removed by corrections for the temperature as measured by thermistors outside the ionisation chamber.

The uncertainty in the ratio of IAEA to ARPANSA calibration coefficients may be obtained from the uncertainty estimates of each laboratory. However, we remove the uncertainty of 0.35% for *W*_*air*_*/e* (twice) as this is correlated. The uncertainty in the ratio is then 0.74% (*k* = 1), and we conclude that the laboratories agree at approximately the *k* = 1 level for all of the beams measured.

## Conclusion

A bilateral comparison between the IAEA and ARPANSA laboratories was conducted showing a high degree of agreement between the calibration coefficients and the radiation qualities established in both laboratories.

The results of this comparison can be used to update the KCDB [13] and promote traceability of radiation dosimetry in medical imaging. This provides the basis for the requirement that testing equipment is calibrated to a traceable standard in RPS S-2 [11] and the requirement that dosimetry equipment must be calibrated in RPS C-5 [10].

## Data Availability

All measurement data can be obtained by contacting the corresponding author.
